# Efficacy and safety of cutting therapy in the treatment of migraine: A protocol for systematic review and meta-analysis

**DOI:** 10.1097/MD.0000000000028084

**Published:** 2021-12-17

**Authors:** Wenping Guo, Hongguang Jin, Yiqiang Wang, Xing Zhu, Guanwei Zhang, Te Wang, Chunhui Fan, Yongsheng Huang

**Affiliations:** a Changchun University of Chinese Medicine; Changchun, Jilin, China; b Affiliated Hospital of Changchun University of Traditional Chinese Medicine; Changchun, Jilin, China.

**Keywords:** cutting therapy, meta-analysis, migraine, protocol, systematic review

## Abstract

**Background:**

Migraine is a chronic paroxysmal neurovascular disease in which pain on one or both sides of the head is the main manifestation and is accompanied by other neurological manifestations. Clinical practice has shown that cutting therapy as a complementary alternative medicine can play a role in relieving migraine attacks. However, there is no consensus on the efficacy of cutting treatment in the treatment of migraine. The aim of this study was to conduct a meta-analysis to systematically evaluate the efficacy and safety of cutting therapy in the treatment of migraine.

**Methods:**

First, databases were searched for relevant literature. The main databases we searched were PubMed, the Web of Science, MEDLINE, Embase, Cochrane Library, the Chinese National Knowledge Infrastructure, the Chinese Science Journal Database, Wanfang Data, and the Chinese Biomedical Literature Database. The keywords searched were “cutting treatment,” “ traditional Chinese medicine cutting treatment,” and “migraine.” The search was conducted from inception to November 2021. Second, 2 reviewers independently completed the process of study selection, data extraction, risk of bias assessment and data synthesis in strict accordance with the Preferred Reporting Items for Systematic Reviews and Meta-analyses Protocols statement guidelines. Finally, we will use Review Manager Version 5.3 software for meta-analysis.

**Results:**

This study will provide the most recent evidence related to the treatment of migraine by cutting therapy.

**Conclusion:**

The results of this systematic evaluation will provide an objective evidence-based framework for judging the effectiveness and safety of cutting therapy in the treatment of migraine.

## 1 Introduction

In today's society, migraine has become a common and frequently occurring clinical disease. A survey shows that more than 1 billion people suffer from migraine every year, and there is no difference in the incidence of migraine across various regions. The World Health Organization lists migraine as the sixth most common disabling disease in the world.^[1,2]^ Therefore, it is of great significance to actively intervene in migraine attacks and prevent migraine recurrence when possible. The onset of migraine is paroxysmal. When it occurs, the patient experiences severe pain on one or both sides of the head, accompanied by photophobia, aversion to sound, nausea and other symptoms.^[2,3]^ The common existing treatment methods, such as analgesia and vasodilation, have little effect on migraine and have obvious side effects.^[4]^ Cutting therapy is a treatment method based on the theory of traditional Chinese medicine and is guided by meridians and acupoints. As a complementary and alternative medicine, cutting therapy has shown good curative effects on migraine. However, there is still a lack of consensus on the efficacy of cutting therapy in the treatment of migraine.

Therefore, this study will systematically review and analyze the existing literature on the treatment of migraine with cutting therapy to evaluate its efficacy and safety and to provide objective evidence for clinical practice.

## 2 Methods

### 2.1 Study registration

We have been registered on the International Registration Platform for Systematic Reviews and Meta-Analysis Programs (https://inplasy.com/inplasy2021-6-0057/.) and registration number is INPLASY2021110029. This protocol will be strictly implemented with reference to the preferred reporting items in the Guideline on Systematic Review and Meta-Analysis Protocol Statement.^[5]^

### 2.2 Inclusion criteria

#### 2.2.1 Types of studies

All randomized controlled trials of cutting therapy for migraine were included in our study, did not consider the blinding method or whether the allocation was concealed in the research reported by the included articles. Our study was not limited by publication year and region, but the language of the literature was limited to Chinese and English.

#### 2.2.2 Types of participants

All patients with a clinical diagnosis of migraine were included in our study, regardless of whether they had an acute or chronic attack, a first attack or a relapse, and regardless of their gender, age, race, belief, region, economic status and education.

#### 2.2.3 Types of interventions

The interventions for the control group were conventional Western medicine treatment (e.g., analgesia, vasodilation), placebo therapy, or psychotherapy. The experimental group was given cutting therapy. In this study, the number of cuts and the frequency of cuts were limited.

#### 2.2.4 Types of outcome measures

1. The primary outcome measures were headache symptom score and headache severity visual analog scale score.
2. The secondary outcome measures were sleep condition, diet, and mental health status.

### 2.3 Exclusion criteria

Repeated published papers;

1. retrospective studies on cutting treatment of migraine;
2. agreements, conference papers, abstracts, nonfull text, and personal reports on experiences with cutting treatment for migraine.

### 2.4 Search strategy

The main databases we searched included PubMed, Web of Science, MEDLINE, Embase, Cochrane Library, the Chinese National Knowledge Infrastructure, the Chinese Science Journal Database, Wanfang Data, and the Chinese Biomedical Literature Database. The search time was from database establishment to November 2021. The content of our search was literature related to the efficacy and safety of cutting therapy in the treatment of migraine, including clinical observations and clinical trials. The keywords searched were “cutting treatment,” “traditional Chinese medicine cutting treatment,” and “migraine.” To avoid data loss, we manually searched the references of the articles that met the criteria. We searched only for publications in 2 languages, Chinese or English, regardless of the quality of the publications.

### 2.5 Study selection and data exaction

#### 2.5.1 Study selection

We will use Endnote X9 (Thomson Corporation, Stanford, CA) to process all the retrieved literature and to delete the repeated articles. We will also draw a flowchart of the screening process to help the screening process go smoothly. After screening is completed, we will combine the inclusion criteria, carefully assess all eligible articles, and extract the data. This process will be performed independently by 2 researchers. If any disagreements occur, they will be resolved by discussion with a third investigator.

#### 2.5.2 Data exaction

We will perform this process using consistent data extraction criteria. This process will also be performed independently by 2 researchers, and in case of inconsistencies in the content-extraction process, discussion with a third researcher will be required. The information extracted for this study mainly will include the basic information of the included study, the basic information of the participants, the intervention methods for migraine, and the outcome indicators.

### 2.6 Risk of bias assessment

The risk of bias for each study will be assessed with the Cochrane Collaboration's tool, and this process will be performed independently by 2 researchers. When the 2 researchers disagree, a third researcher will make the final decision. The main areas we will assess include randomized sequence generation; allocation sequence concealment; blinding of participants and personnel; blinding of outcome assessment; incomplete outcome data; selective outcome reporting; and other biases. Upon completion of the assessment, each assessed area will be classified as having a low, high or unclear risk of deviation.

### 2.7 Data synthesis and analysis

#### 2.7.1 Data synthesis

This study will use Review Manager Version 5.4 software (The Cochrane Collaboration, Copenhagen, Denmark) for meta-analysis. The effects of continuous variables will be expressed as the mean difference (MD) or standardized mean difference. The efficacy of binary variables will be calculated using the hypothetical risk ratio (RR) or odds ratio (OR), setting 95% as the confidence interval (95% CI). Heterogeneity will be determined with the *I*^2^ test. When *I*^2^ ≤ 50% and *P* > .1, this indicates that our study is uniform, and the fixed effect model will be selected for meta-analysis. When *I*^2^ > 50% and *P* < .1, this indicates that the difference is statistically significant, and a random-effects model will be used.

#### 2.7.2 Subgroup analysis

Subgroup analysis will be performed if data are available and sufficient, such as different intervention time, and different stages of migraine.

#### 2.7.3 Sensitivity analysis

To judge the robustness and stability of the review results, we will conduct a sensitivity analysis. Through sensitivity analysis, we will remove low-quality studies with small sample sizes, high risk of bias, or missing data.^[6]^

#### 2.7.4 Reporting biases assessment

Funnel plots will be used in this study to assess publication bias that may occur in research with >10 studies.^[7]^

#### 2.7.5 Evidence quality assessment

We will evaluate the quality of the evidence from Grading of Recommendations Assessment, Development, and Evaluation. The quality of evidence was divided into 4 levels: high, medium, low, and very low.

#### 2.7.6 Missing data management

In the event that relevant data is missing, we will contact the authors by e-mail or telephone. When the author cannot be contacted or if accurate data cannot be obtained after contacting the author, the study will be excluded.

### 2.8 Ethics and dissemination

Our study involves neither patient recruitment nor animal experimentation and therefore dose not require ethics committee approval.

## 3 Discussion

With the rapid development of society, migraine has become a major problem affecting people's ability to work and quality of life. There are no specific biological markers or diagnostic imaging characteristics that can predict the occurrence of migraine.^[8]^ Current studies have shown that sleep disorders, anxiety, depressive states, and estrogen levels are closely related to the occurrence of this disease.^[9–11]^ In the treatment of migraine, analgesic-based interventions are the most commonly used therapies and include drugs such as ibuprofen and opioids; however, with ongoing clinical observation, the efficacy of these classes of drugs and their side effects have raised questions and concerns.^[12,13]^ Long-term clinical practice has confirmed that cutting therapy, as a complementary alternative therapy, has shown good results in the treatment of migraine. This therapy is a new application based on the guidance of the theory of acupuncture and moxibustion acupoints in traditional Chinese medicine. However, there is no consensus on the efficacy and safety of this therapy. In this study, we have collected and analyzed clinical research data on the treatment of migraine with cutting therapy; our goal is to summarize the efficacy and safety of this treatment and to provide evidence-based guidance for its clinical application.

## Author contributions

**Conceptualization:** Wenping Guo, Yongsheng Huang.

**Data curation:** Wenping Guo, Guanwei Zhang, Te Wang.

**Formal analysis:** Te Wang, Chunhui Fan.

**Investigation:** Yiqiang Wang, Xing Zhu.

**Methodology:** Hongguang Jin, Yiqiang Wang, Xing Zhu.

**Resources:** Wenping Guo, Hongguang Jin.

**Software:** Wenping Guo.

**Supervision:** Yongsheng Huang.

**Validation:** Hongguang Jin.

**Visualization:** Hongguang Jin.

**Writing – original draft:** Wenping Guo, Hongguang Jin.

**Writing – review & editing:** Wenping Guo, Hongguang Jin.
